# Ladder-Shaped Ion Channel Ligands: Current State of Knowledge

**DOI:** 10.3390/md15070232

**Published:** 2017-07-20

**Authors:** Yuri B. Shmukler, Denis A. Nikishin

**Affiliations:** Group of Embryophysiology, N.K. Koltzov Institute of Developmental Biology, Russian Academy of Sciences, 26, Vavilov st, 119334 Moscow, Russia; d.nikishin@idbras.ru

**Keywords:** ciguatoxin, maitotoxin, brevetoxin, brevenol, voltage-gated Na^+^-channel, thylakoid membrane, light-harvesting complex II

## Abstract

Ciguatoxins (CTX) and brevetoxins (BTX) are polycyclic ethereal compounds biosynthesized by the worldwide distributed planktonic and epibenthic dinoflagellates of *Gambierdiscus* and *Karenia* genera, correspondingly. Ciguatera, evoked by CTXs, is a type of ichthyosarcotoxism, which involves a variety of gastrointestinal and neurological symptoms, while BTXs cause so-called neurotoxic shellfish poisoning. Both types of toxins are reviewed together because of similar mechanisms of their action. These are the only molecules known to activate voltage-sensitive Na^+^-channels in mammals through a specific interaction with site 5 of its α-subunit and may compete for it, which results in an increase in neuronal excitability, neurotransmitter release and impairment of synaptic vesicle recycling. Most marine ciguatoxins potentiate Na_v_ channels, but a considerable number of them, such as gambierol and maitotoxin, have been shown to affect another ion channel. Although the extrinsic function of these toxins is probably associated with the function of a feeding deterrent, it was suggested that their intrinsic function is coupled with the regulation of photosynthesis via light-harvesting complex II and thioredoxin. Antagonistic effects of BTXs and brevenal may provide evidence of their participation as positive and negative regulators of this mechanism.

## 1. Introduction

Ciguatera fish poisoning (CFP) is one of the most important non-bacterial disease nowadays, which is induced by consummation of fish contaminated by specific dinoflagellates of *Gambierdiscus* lineage [1]. Furthermore, it is the most prolonged marine intoxication [2], which sometimes can be fatal. The basic symptoms of CFP usually involve short-term gastrointestinal disorders (such as nausea, vomiting, diarrhea and so on), cardiovascular and more long-lasting neurological pathologies, such as dysesthesia (reversal of cold and hot sensation) (see review [3]).

Neurotoxic shellfish poisoning (NSP) [4] shows symptoms similar to CFP, although this has less severe forms in humans. However, it is highly dangerous for big marine animals, such as bottlenose dolphins [5,6]. In this case, the source of toxins is another unicellular dinoflagellate microalga *Karenia brevis.* These pathologies—CFP and NSP—and the toxins evoking them are often considered together because on the one hand, the toxins share closely similar ladder-shape polycyclic ether structures and on the other hand, they are well known as the unique natural activators of the same target, namely voltage-gated Na^+^ channels (VGSC) [7,8,9]. The specificity of the target for these toxins determines the similarity of pathologies and occurrence of VGSC in all excitable cells, which is the reason for the variety of symptoms caused by these toxins. In particular, cold allodynia is considered as the most characteristic symptom of CFP, which is pain sensation evoked by normally non-painful temperature stimuli [10,11].

Interestingly, the research field under consideration has evolved from a set of quite simple facts and ideas through step-by-step sophistication similar to the growth of areal of microalgae, producing the compounds of interest. At first, these dinoflagellates were found within certain limited areas in tropical waters, although their habitat seems to be much more extensive nowadays. Similarly, the initial interest in ciguatoxins (CTX) and brevetoxins (BTX) was preconditioned by medical reasons, associated with NSP and CFP, instead of being as the result of their established role as the specific ligand. Nowadays, the list of toxins and spectrum of their effects have expanded with the expansion of the geographic borders of their distribution. Therefore, nothing is left from the original simplicity of the problem.

All of the abovementioned points form the ground for the unfailing and thorough interest in the physiological, ecological and biomedical aspects of the problem of the toxins produced by these dinoflagellates. It is reflected in the number of already published studies and basic reviews (see [8,12]). Nevertheless, continuously changing practical needs and widening experimental abilities has brought up new problems and facts, which deserves the attention of specialists in this field of research. In particular, the evaluation of annual number of patients suffering of ciguatera counted approximately 20,000 people a decade ago [13], while the more recent works quote the incidences *per annum* of 50,000 to 500,000 [3,14,15,16]. At the same time, some ladder-shape compounds are possible candidates for drug development.

Therefore, the present review mainly pays attention to the data published during the last decade. In the present review, we examined the following issues:

(i) current taxonomy of these harmful algae;
(ii) geographic distribution of the species under consideration in the new areas over the world;
(iii) current state of knowledge of the spectrum of toxic compounds, produced by lineage *Gambierdiscus* and genus *Karenia*;
(iv) toxins’ effects and their physiological mechanisms, especially interactions with ion channels;
(v) intracellular functions of these substances in the marine algae *Gambierdiscus* and *Karenia*, which have been poorly studied until now.

## 2. Actual State of Taxonomy

Recently, significant changes were amended and supplemented in the taxonomies of both genera.

Among dinoflagellates of *Gamberdiscus* genus species, *G. toxicus* was the first to be described (see [3] for the history of description) and remain the most important as the source of the marine intoxication, namely ciguatera. The important changes in the taxonomy of lineage *Gambierdiscus* were applied even when compared to recent publications in the field (see [17]). Initially, the *Gambierdiscus* genus included two easily distinctive morphotypes: globular and discoid (or lenticular), which are also differentiated by phylogenetical molecular methods [17,18,19,20,21,22]. According to this morphological feature, lineage *Gambierdiscus* has been split into two genera: the original name reserved for the species with lenticular shapes, whereas a new genus *Fukuyoa* (gen. nov.) applies to the globular species [23].

During the preparation of this review, a description of one new species was reported. The new tropical epiphytic dinoflagellate species, *Gambierdiscus honu* sp. nov., was obtained from macroalgae. The phylogenetic analyses supported the unique description [24].

Thus, the Gambierdiscus genus, characterized by lenticular morphology, consists of 15 species at the present moment (*G. toxicus*, *G. belizeanus*, *G. pacificus*, *G. australes*, *G. polynesiensis*, *G. caribaeus*, *G. carolinianus*, *G. carpenteri*, *G. excentricus* (newly described) [25], *G. scabrosus* [26], *G. silvae* [27]; *G. balechii* [28], *G. cheloniae* [29], *G. lapillus* [30] and *G. honu* sp. nov. [24]).

The new genus *Fukuyoa* F. Gomez, D. Qiu, R. M. Lopes and S. Lin, designated by globular forms, includes *F. yasumotoi* (earlier—*Gambierdiscus yasumotoi*), *F. ruetzleri* (earlier—*Gambierdiscus ruetzleri*, [17]) and the newly described species, *F. paulensis* F. Gomez, D. Qiu, R. M. Lopes and S. Lin [23]. Although the designation of *Fukuyoa* genus is well-established as the new taxon for globular species of *Gambierdiscus* lineage, the specific limits inside the genera remain contradictory and imprecise [21,31,32].

Genus *Karenia* was named for Dr. Karen A. Steidinger in 2001. Correspondingly, the dinoflagellates previously known as *Gymnodinium breve* and *Ptychodiscus brevis* have become *Karenia brevis.* This unicellular marine alga is responsible for the coastal infestations termed “red tides”, which affects Gulf of Mexico coasts. These organisms are the source of various toxins, including the eponymously named brevetoxins. Together with *K. brevis*, a number of species in the *Karenia* genus have been reported to cause severe blooms, including *K. mikimotoi* (formerly *Gyrodinium aureolum*, *G*. cf. *aureolum*, *G*. *nagasakiense* and *G*. *mikimotoi*). Blooms of this species caused the mortality of fish and invertebrates in Hong Kong and in Japan [33].

The *Karenia* genus also includes *K. selliformis*, *K. longicanalis*, *K. papilionacea* and *K. digitata* [29,34,35,36,37] in addition to a recently described new species *K. brevisulcata* [38].

## 3. New Data on the Geographic Distribution of *Gambierdiscus* Lineage and Genus *Karenia*

The pathogenesis of CFP is caused by compounds, which were originally named gambiertoxins after the place of its discovery by the expedition near Gambier Islands in French Polynesia [39]. After this, these toxins of *Gambierdiscus* species were given the name of ciguatoxins and were specially marked with prefixes: Р for Pacific, С for Caribbean and I for Indian ocean. These prefixes are assigned due to the peculiarities of their structures in these distant areas (see [40]). Moreover, even if the occurrence of *G. toxicus* is generally restricted to the tropical and subtropical coral reef areas [3], a spread of ciguatoxic fish has been observed in previously unaffected regions in recent years.

Initially, the NSP habitat was associated with Gulf of Mexico exclusively, where the brevetoxins (BTX) produced by the dinoflagellate *Karenia brevis* are responsible for high fish and marine mammal mortality [41]. Nowadays, it also spreads along the East coast of the United States. In New Zealand, BTXs have begun to threaten shellfish farming since the outbreak in 1990s [42].

The problem of CFP and NSP dissemination has two aspects. The first is the medical problem related with the globalization of fishing industry as well as contaminated fish and seafood export all over the world. The absence of external indications, limited techniques of determination as well as thermostability of CTXs and BTXs have become concomitant factors causing local CFP and NSP outbreaks in countries that are located distant from the common habitats of these algae [40]. Tens of ciguatera cases caused by consummation of contaminated fish were already recorded in various countries of Europe and America [16,43]. It has to be taken into account that the area of the occurrence of ciguatera fishes may be wider than the harmful algae habitat itself because of the possibility of long distance migration of contaminated fish.

There are two main causes of the spread of these harmful marine algae by themselves. Firstly, the global warming has contributed to the emergence of dinoflagellate species in previously subtropical and even temperate regions [16]. Secondly, it has been found that this temperature range itself may be wider than thought [43]. Finally, the anthropogenic factor plays an important role in the distribution of these microalgae. The expansion of the aquatic area in temperature range combined with the role of contemporary transport seaways has increased the area that is appropriate for the reproduction of *Karenia* and *Gambierdiscus*. This provides the possibility for the fast distribution of these species and changes in areas as the result of the backrush of ballast waters [43].

Global warming and the associated processes of the expansion of species of genus *Gamberdiscus* in East Atlantic and Mediterranean increase the threat of CFP in Europe nowadays due to microalgae invasion towards its coasts [40,44]. The tropicalization scenario of the Mediterranean Sea [45] is supported by the increase in the sea surface temperature in the region [46] also in addition to probable migration through the Suez Canal or invasion via the Strait of Gibraltar. Correspondingly, there is partially supported information on the positive immunobead assay for the presence of CTXs in warmer eastern part of Mediterranean near the Israeli coast [47]. Simultaneously, the data were obtained related to the presence of microalgae of interest in the colder waters near Crete and Canary Island [48,49,50,51]. Furthermore, two outbreaks of CFP were recorded in Madeira Archipelago 260 miles north of the Canary Islands [40].

Considering the impact of climate change on ciguatera [52], it was suggested that aside from the increase in the sea surface temperature (SST), the extended periods during which the water temperature is high may depress the occurrence of ciguatera. Such a model presents a more sophisticated way of climate change affecting ciguatera compared to a simple increase in CFP with the rise of SST. The extrapolation of this model to the Mediterranean waters suggest the importance of monitoring the sea surface temperature in addition to *Gambierdiscus* spp. populations in order to assess a possible onset of ciguatera in the Mediterranean Sea and Macaronesia in relation to global warming [40].

The microalgae *Fukuyoa paulensis* were found in the western part of Mediterranean Sea on the coast of the island of Formentera (Balearic Islands), which is cooler than its eastern basin. It was cultured and characterized using morphological and molecular methods in addition to toxin analyses. Molecular analysis has shown that this *F. paulensis* strain contains large-subunit ribosomal RNA (LSU rDNA) sequences, which are identical to New Zealand, Australian and Brazilian strains [53].

The cases of CFP in Asia include the outbreaks in China, Japan, Taiwan and Malaysia [44,54]. The CFP in Australia was linked to Pacific ciguatoxin-1B and initially occurred in connection with the fish from tropical Queensland and Northern Territory waters. However, the later locations of ciguatera fish were shifted about 400 km to the south to New South Wales coastal waters [55,56]. It is suggested that the increase in ocean water temperature and the intensification of the East Australian Current are the reasons for the shift of this CFP threat into more southern Australian waters [57]. In Australian waters, *Gambierdiscus belizeanus*, *Fukuyoa yasumotoi* [31], an unknown *Gambierdiscus* sp. genotype (A213) [58], *G. carpenteri* [54] and *G. lapillus* sp. nov. [30] were found.

Outside of the original area of *Karenia brevis* in the coasts of Gulf of Mexico and in Florida [59], the first recorded major bloom of the *Karenia* species in New Zealand occurred in 1992/93 along the coast of Northland. It was accompanied with the outbreak of illness with the symptoms of neurotoxic shellfish poisoning (NSP) [60,61,62]. The identity of the causative organism was determined as *K. mikimotoi* [62]. *K. mikimotoi* also formed massive blooms in the coastal waters of the temperate regions in Norwegian, Swedish and Scottish water as well as in East Asia, especially west Japan [33].

Along with the geographic expansion, there has been growth in the list of host-species in which these harmful algae are being detected. Among herbivorous fishes and molluscs [43,63], possible CTXs were discovered in echinoderms—two starfish species *Ophidiaster ophidianus* and *Marthasterias glacialis* from Madeira and Azores archipelagos in the northwestern Moroccan coast. The amount of toxin was significant, which confirms the importance of this theme [64]. CTX-like compounds were found in giant clams and sea urchins [65] in addition to various shellfishes [66].

## 4. Common Problems of the Compounds, Produced by *Gambierdiscus* and *Karenia*

Ciguatoxins (CTXs) and brevetoxins (BTXs) are the two main suites of marine dinoflagellate derived polyether neurotoxins. Their main common target is the voltage gated Na^+^-channels (VGSC), which are present on membranes of all excitable cells, such as the skeletal muscles, nervous and heart tissues. These channels exist in a number of isoforms, which are differentially expressed depending on species and tissues. The physiological and pathological, especially neurological, effects of both CTXs and BTXs are the results of their interaction with the α-subunit of VGSC site 5 [7,8,9,67]. The voltage-sensitive α-subunit consists of four homologous domains, which each contain six transmembrane segments that form the pore as well as facilitating voltage-sensing and ligand binding. The α-subunit of VGSC of mammalian isoforms Na_v_1.1–1.9 can be associated with the β-subunit [68].

Both suites of toxins are lipid-soluble, thermally-stable polyether ladder-shaped polycyclic ethereal compounds. CTXs and BTXs are hard to detect because they do not have clear marking properties, such as flavor or taste. The only way to exclude the possibility of CFP and NSP is to avoid the use of potentially hazardous fish and sea food.

Only recently, some new relatively fast and safe methods of such toxins detection were introduced. One of them represents a simplified procedure for extracting polyether toxins from *Gambierdiscus* and *Fukuyoa* spp. based on the CTX rapid extraction method (CREM). Fractionated extracts are analyzed using a functional bioassay, which records the intracellular Ca^2+^ changes in response to sample addition in SH-SY5Y neuroblastoma cells [63]. Using the neuroblastoma cell-based assay (CBA-N2a) of the extracts from the marine dinoflagellates *Gambierdiscus*, the presence of CTX-like compounds in giant clams *Tridacna maxima* and sea urchins *Tripneustes gratilla* were shown, which suggests a second bio-accumulation route for CFP toxins in the ciguatera food chain along with the standard one that includes the herbivorous fishes [65].

A fluorescence-based receptor binding assay (RBA(F)) was developed to provide a method of screening fish samples for CTXs to avoid need to use of radioisotopes. The new assay is based on the competitive binding of CTXs and fluorescently labeled brevetoxin-2 (BODIPY^®^-PbTx-2) for VGSC site 5. The RBA(F) takes approximately two hours to perform. As a widely used CBA-N2a assay requires 2.5 days to complete, this RBA(F) is far more satisfactory for the practical needs in this field [69].

The chemical and physiological data on the main active substances CTXs and BTXs, produced by dinoflagellates *Gambierdicus* and *Karenia* are shown below in addition to several others, such as gambierol, maitotoxins and brevenal.

## 5. Compounds, Produced by *Gambierdiscus* and Their Physiological Effects

The suite of the substances produced by the microalgae of the *Gambierdiscus* lineage is large in number and varied. Together with CTXs, there is a number of compounds with various chemical and pharmacological characteristics, including those with radically different properties compared to CTXs themselves.

### 5.1. Ciguatoxins (CTXs)

**Figure 1:**
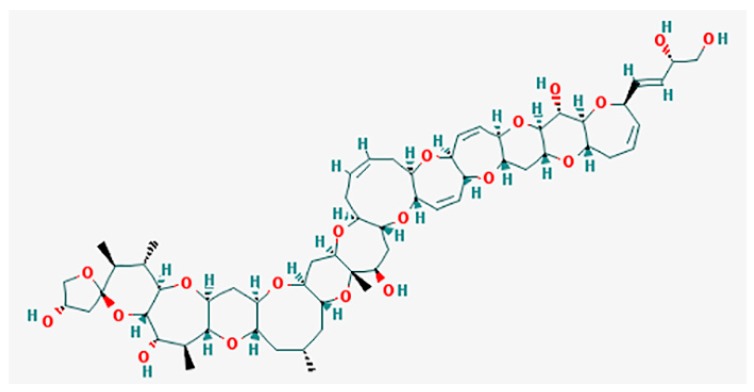
Structure of Pacific ciguatoxin-1 (P-CTX-1) [72].

CTXs (Figure 1) considered as the main cause of CFP are formed by 13–14 heterocyclic rings and comprise more than twenty congeners to date, identified in Pacific region. Furthermore, there are at least 12 known congeners among Caribbean and tropical Atlantic fish [3,8,57,70]. Despite the widespread occurrence of *Gambierdiscus* spp. and CFP, only several strains (*G. toxicus, G. polynesiensis, G. australes*, *Fukuyoa* (formerly Gambierdiscus) *yasumotoi*, *G. pacificus* and *G. belizeanus*) have been confirmed as the producer of CTX by liquid chromatography-mass spectrometry (LC/MS) analysis or receptor binding assay (see [71]).

The original concept suggests that the toxins evoking the ciguatera syndrome are the final product of a long sequence of synthesis steps. It starts with synthetic processes in the dinoflagellate *Gambierdiscus toxicus* and continues via the bioaccumulation and metabolism of precursor toxins along the food web. The latter includes marine invertebrates, such as shellfishes [43], herbivorous fishes and large predatory fishes [17,39,54,70]. The precursors of ciguatoxins contain spiroketal (also known as spiroacetal). When coupled with carbon 52, these precursors undergo an acid catalyzed spiroisomerization during their passage through marine food chains in an acidic medium (e.g., in the fish stomach) [73]. The network of CTX transformations starts from CTX-4A, which, on the one hand, by sequential hydroxylation converts into CTX-2 and CTX-4, and, on the other hand, it transforms into CTX-4B by hydrogenation. In turn, the latter transforms to CTX-3 and CTX-1 by two consequent stages of hydroxylation. [73].

Remarkably, the biotransformation of CTX-4В (formerly the gambiertoxin GTX-4B) into CTX-1 leads a 10-fold increase in toxin potency [74,75]. Many compounds mentioned in the metabolic Scheme 1 are present in the extracts from *G. polynesiensis* culture, including CTX3C, 49-epi-CTX-3C (synonym: CTX-3B), CTX-4A and CTX-4B [57]. It was suggested that toxins can accumulate to a greater extent in adult and larger fishes [73], similar to domoic acid that evokes paralytic shellfish poisoning [76]. However, verification of this hypothesis, especially in sharks, shows no detectable levels of CTX-1, -2 and -3 [77,78].

The most powerful ciguatoxin P-CTX-1 has attracted the most attention from researches. It is noted that P-CTX-1 is a well-known ligand of VGSCs and competitively binds to these native channels in the brain, heart and skeletal muscle of rat as well as the marine teleosts in the presence of [^3^H] PbTx-3 [79]. An exhausting study of the electrophysiological effects of P-CTX-1 on VGSC isoforms Na_v_1.1–1.9 was carried out in HEK293 cells. P-CTX-1 has shown the ability to influence all Na_v_ isoforms studied but with peculiar effects on Na^+^ channels. First, P-CTX-1 reduces the activation threshold of the two key isoforms, Na_v_1.8 and Na_v_1.7 channels, and lengthens their active periods, contributing to the activity of nociceptive neurons [68].

The most prominent CFP symptom, allodynia, is the type of temperature dysesthesia. Specifically, it is the acute pain sensation similar to that of an electric shock when touching cold water. This symptom, evoked by both BTX and CTX, means close similarity or identity of physiological mechanisms of these toxins. On the other hand, it is highly probable that such specific pathology is associated with the disturbances of VGSC function [10,11].

It is suggested that the source of the cold hypersensitivity is a specific peptidergic neuron population in the dorsal root ganglia, involving various voltage-gated channels [10]. The activation of Na_v_1.6 and Na_v_1.7 in HEK293 cells by P-CTX-1 contribute to the peripheral sensitization to dynamic cold stimuli in Aδ-fibers, while the effects of P-CTX-1 on Na_v_1.8 are especially important for the majority of С-fiber nociceptors, which induce allodynia [68,80]. Closely similar results were obtained with intra-plantar injection of P-CTX-1 in mice, which elicited cold allodynia by targeting specific primary sensory neurons. These also involved both tetrodotoxin-resistant, transient receptor potential cation channel, subfamily A, member 1 (TRPA1)-expressing peptidergic C-fibers and tetrodotoxin-sensitive Aδ-fibers. P-CTX-1 does not directly open heterologously expressed TRPA1, but a Na^+^-channel activation by P-CTX-1 is sufficient to drive TRPA1-dependent Ca^2+^ influx that is responsible for the development of cold allodynia [10].

Interestingly, the gastrointestinal sensory disturbances evoked by P-CTX-1 are mediated predominantly by the same Na_v_1.8 isoform as in the case of cold and mechanical stimuli effects. Therefore, the various in vivo effects induced by P-CTX-1 appear to be driven predominantly by Na_v_1.6, Na_v_1.7 and Na_v_1.8. It is concluded that the behavioral effects associated with ciguatera reflect the expression pattern of Na_v_ isoforms in peripheral nerve endings [68].

P-CTX-1 in nanomolar concentrations is able also to block voltage-gated K^+^-channels in rat myofibrils [81] and sensory neurons [82], which are opposite to the effects of CTX-3C in K^+^- and Ca^2+^-channels of mouse cortical neurons (see below) [83].

P-CTX-1 is able to persist in mouse peripheral nerves from several hours to two months after exposure, which may explain the long duration of neurological disturbances. Evidence has shown that the persistence of P-CTX-1 in peripheral nerves inhibits both axonal regrowth after injury and reduces other capacities of peripheral neurons, resulting in delayed functional recovery [84].

P-CTX-1 remarkably reduced mouse δ- and θ-electroencephalography (EEG) activity in 2 h, which returned to normal in 6 h after a single exposure. However, a second exposure to P-CTX-1 induced a further reduction in EEG activities and a 2-week delay in returning to baseline EEG values. Ciguatoxicity was detected in the brain some hours after the first and second exposure by the mouse neuroblastoma assay. The spontaneous firing rate of a single motor cortex neuron was reduced significantly in contrast to peripheral neurons. The expression profile study of neurotransmitters using liquid chromatography-tandem mass spectrometry revealed an imbalance between excitatory and inhibitory neurotransmitters in the motor cortex [85].

P-CTX-1 increases neuronal spontaneous activity and responses to visceral pain stimuli in the rat anterior cingulate cortex (ACC). It induced synaptic potentiation in the medial thalamus (MT)-ACC pathway. Further studies have shown an enhanced expression of Na^+^-channels in astrocytes. It is suggested that astrocytes may be activated on ciguatera poisoning in vivo, which is confirmed by an activation of gap junction protein connexin 43 and transporter 2 of excitatory amino acids. However, neurotoxicity and reactive astrogliosis were not found in ACC 7 days after P-CTX-1 exposure. These results provide evidence in favor of neuronal excitotoxicity in the P-CTX-1 brain cortex in vivo in addition to an important role of cortical neurons and astroglia in acute ciguatera poisoning [86]. At the same time, nanomolar P-CTX-1 intradermally administered to humans leads to somatosensory symptoms of ciguatera poisoning, in particular pruritis and cold allodynia, showing the direct effect onto primary sensory afferents [11].

Ciguatoxin is also associated with actions on other systems, such as immune cells, which are not traditionally viewed as excitable. The genomic response to mouse males’ exposure to P-CTX-1 shows the involvement of the genes of cytokine signaling, proteasomic complex and ribosomal function. The serum protein analysis showed small but significant changes in 6 of 60 proteins assayed: Ccl2, Ccl12, CD40, IL-10, leptin and M-CSF. Generally, the gene expression was consistent with a Th2 immune response [87].

The global transcriptional response in the mouse brain to a symptomatic dose of P-CTX-1 shows the enrichment for complement/coagulation cascades and metabolism of xenobiotics. Many immediate early genes, such as Fos and Jun, were down-regulated, although others associated with stress were up-regulated, such as glucocorticoid responsive genes. The pathologic activity of the complement/coagulation cascade has been shown in patients suffering from a chronic form of ciguatera poisoning, including the up-regulation of genes in the brain, liver and blood, such as FK506 binding protein 5, polymeric immunoglobulin receptor 3 precursor and mitogen-activated protein kinase kinase. At the same time, hematopoietically expressed homeobox and interleukin 1β were down-regulated [88].

P-CTX-1B coincides with P-CTX-1 in the backbone structure but differs in some radicals. P-CTX-1B-evoked Na^+^-influx significantly increases catecholamine release from presynaptic nerve terminals and impairs synaptic vesicle recycling, which contributes to ciguatera symptoms [67]. CTX promoted processes were suggested to be caused by Ca^2+^ entry either through the reverse mode activation of the Na^+^/Ca^2+^ exchanger or via direct mobilization of Ca^2+^ from internal stores [81]. The rise in the intracellular Ca^2+^-levels could in turn stimulate exocytosis of synaptic vesicles [89]. P-CTX-1B also stimulates the catecholamine secretion from the bovine chromaffin cells [90].

P-CTX-2 is a less-oxidized form of P-CTX-1, which exists as a diastereomer of P-CTX-3 [91]. Electrophysiological recordings from rat dorsal horn neurons show that P-CTX-2 increased neuronal responses to innocuous and noxious cooling as well as to the low-threshold but non-noxious mechanical ones. The latter response was probably caused by the P-CTX-2 selective sensitization, which might be dependent on Na_v_1.8-positive Aδ-fiber low-threshold mechanoreceptors and/or C-fiber low-threshold mechanoreceptors [92]. The mechanical sensitization was a transient effect, while cold hypersensitivity persisted for several hours. The injection of the Na_v_1.8. antagonist A803467 reduced both mechanical and cold hypersensitivity [93]. Unlike other Na^+^ channels, the inactivation kinetics of Na_v_1.8 are resistant to cold, which identified the Na_v_1.8-positive neurons as critical for the detection of noxious cold temperatures [94].

Na_v_1.9 frequently co-localizes with Na_v_1.8 and has recently been identified as a subthreshold amplifier of cold transducer currents. Na_v_1.9 knockout mice exhibit normal temperature discrimination but impaired responses to noxious cooling ramps [95]. P-CTX-2 enhanced the neuronal responses to innocuous and noxious cold stimulation in the absence of any obvious changes in central neuronal excitability or coding of other modalities. Cold hypersensitivity induced by ciguatoxin is dependent on a subset of Na_v_1.8/TRPA1-positive afferents [93].

The nanomolar concentrations of C-CTX-1 induced the up-regulation of Na^+^-channels and the inhibition of K^+^ channels, which produced a variety of functional disorders of human skeletal muscles observed in ciguatera. All these disorders seem to be the result of the subtle balance between ionic currents, intracellular Na^+^ and Ca^2+^ concentrations as well as engaged second messengers [96].

In the pico/nanomolar concentration range, C-CTX-1 dose-dependently shortened the duration of the plateau and the repolarizing phase of the action potential in the auricle of frogs. This effect was suppressed or prevented either by tetrodotoxin or by the muscarinic acetylcholine receptor (mAChR) antagonist atropine. Other mAChR-antagonists also prevented or reversed the effects of C-CTX-1, while acetylcholine (ACh) mimicked the effects of C-CTX-1 [96], because the latter was able to enhance quantal ACh release [97].

Micromolar concentrations of CTX-3C shift the activation potential in the negative direction and the threshold potential to hyperpolarized potentials in all Na^+^-channel isoforms. The toxin significantly accelerated the time-to-peak current in the isoform Na_v_1.2. In higher doses, the toxin also had a similar effect in the isoforms Na_v_1.4 and Na_v_1.5 in HEK293 cells, but it preferentially influences Na_v_1.8, which is involved in pain transducing. Recovery from slow inactivation was delayed in the presence of CTX-3C [98,99]. Using two chimeric constructs, it was determined that the VGSC region conferring high sensitivity to CTX-3C action is located in the D1 or D2 domains [98]. At the same time CTX-3C decreased K^+^ currents in granular cerebellar cells [100]. CTX-3C has been reported neither to modify K^+^-channels in mouse taste cells nor in cortical neurons. Furthermore, CTX-3C did not affect voltage-gated Ca^2+^-channels [83].

The effects of CTX on the invertebrate embryonic development is poorly investigated. Nevertheless, it is known that this toxin has a lethal effect in *Artemia* nauplii [101]. When 0.5 nM of CTX is added to the fertilization of the sea urchin *Paracentrotus lividus*, this triggers a fast and large rise in cell Na^+^ levels, which was eightfold higher than in a control [102]. This suggests the involvement of the same VGSC mechanisms as in adults. Surprisingly, nobody has tried to revise data in this classic model although it gives an opportunity to trace the influence of various substances on the diverse processes of the development [103,104].

In another model of the marine medaka *Oryzias melastigma* at various developmental stages, microinjections of P-CTX-1 decreased viability and evoked hatching failure [105]. Similarly, in fresh water, *Oryzias latipes* microinjections of pictogram amounts of CTX evoked skeletal deformities (spinal curvature) and a reduction in hatching success compared to that in controls [106]. The severity of spinal curvature was dose-dependent and induced forced circular swimming, which eventually resulted in death [107].

Thus, the studies in recent years confirmed the main extrinsic function of CTXs as the activators of VGSC and made some steps towards the disclosure of the mechanisms of the consequences of such effects. Along with the variety of CTXs, the microalgae of *Gambierdiscus* lineage produce some other compounds of similar polycyclic ether structures but differing in their functions. A set of such compounds includes gambieric acids [108], gambierol [109], gambieron [110] and maitotoxins [111,112] (see below).

### 5.2. Gambierol

Gambierol from *G. toxicus* takes part in CFP pathogenesis, inducing neurological symptoms in mice [113]. In contrast to CTXs, it does not affect the voltage-dependent Na_v_ channel function [114,115], although gambierol and gambieric acid A are able to inhibit binding of the brevetoxins to the VGSC [116].

**Figure 2:**
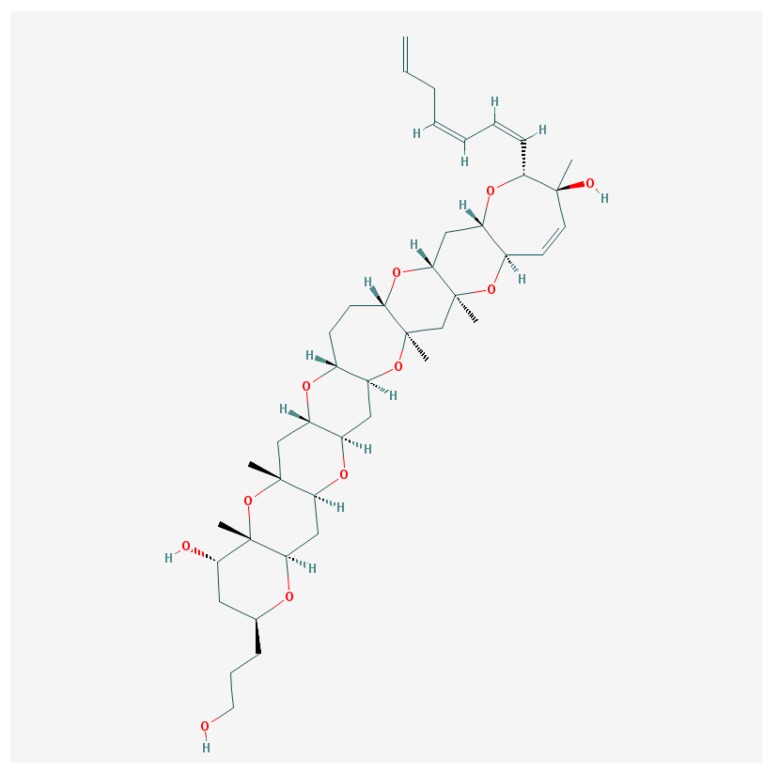
Gambierol structure [119].

The screening of gambierol (Figure 2) effects was performed in the panel of 9 cloned VGSC (Na_v_1.1–Na_v_1.8 mammals and insect Para) and 8 cloned voltage-gated K^+^-channels (VGPC) (mammalian K_v_1.1–K_v_1.6, hERG—K_v_11.1 and Shaker-channels from insects), which were expressed in *Xenopus laevis* oocytes using the two-electrode voltage-clamp. All VGSC were insensitive to gambierol even in micromolar concentrations [115]. At the same time, this compound evoked almost a total block of voltage-dependent K_v_1 and K_v_3 channel subtypes in the nanomolar range of concentrations [115,117,118].

Gambierol and its truncated tetracyclic and heptacyclic analogs share the main crucial elements for biological activity, which are the C28=C29 double bond within the H-ring and the unsaturated side chain. Similar to its truncated analogs, gambierol inhibits K_v_1.2 and K_v_1.3 channels present in resting T-lymphocytes in nanomolar concentrations. Gambierol cytotoxicity in Chinese hamster ovary cells (CHO) is not triggered by an inhibition of the K_v_, but negatively affects the expression of genes involved in cell proliferation and immune response [120,121].

It is most likely that gambierol influences K_v_ channels via a lipid-exposed binding site. Using the ionic current analysis, it was shown that gambierol binds with a high affinity to resting (closed) channel, affecting S6 gate opening and voltage-sensing domain (VSD) movements. The transitions between the resting and the opened channel state first require its dissociation from gambierol, which is facilitated by this state having a much lower affinity for gambierol [120]. Using chimeric channels between K_v_3.1 and K_v_2.1, it was shown that only the S3b–S4 part of the VSD decreased gambierol sensitivity in K_v_3.1 by more than 100-fold [122].

Gambierol has been shown to induce augmentation of spontaneous Ca^2+^ oscillations in cerebrocortical neurons as a consequence of K_v_ channel inhibition. Interestingly, gambierol in these cells produced a concentration-dependent stimulation of neurite outgrowth that was mimicked by 4-aminopyridine, a universal K^+^-channel inhibitor [123], in contrast to the abovementioned inhibitory effect of P-CTX-1 in axotomized peripheral neurons [84].

Gambierol also produces a robust stimulation of the phosphorylation of extracellular signal-regulated kinases 1/2 (ERK1/2). This stimulation is dependent on both N-methyl-D-aspartate (NMDA) and glutamate receptors (mGluRs), because corresponding inhibitors attenuate the response. In addition, the inhibitors of inositol, namely 1,4,5-trisphosphate receptor and phospholipase C, both suppress gambierol-induced ERK1/2 activation, which further confirm the role of mGluR-mediated signaling in the observed ERK1/2 activation [123].

The effects of gambierol and its truncated analogues were also studied in an in vitro model of Alzheimer’s disease obtained from transgenic mice, which expresses amyloid-β accumulation and tau protein hyperphosphorylation. The preincubation of these cells with these compounds resulted in a reduction in the extra- and intracellular levels of Aβ in addition to a decrease in the levels of hyperphosphorylated tau protein. Furthermore, these compounds reduced the level of the NMDA-receptor subunit 2A without affecting the 2B subunit. The involvement of glutamate receptors was further suggested by the blockage of the effect of gambierol on tau protein hyperphosphorylation by glutamate receptor antagonists [124]. It was the first demonstration of the utility of gambierol and its synthetic analogues as chemical probes for understanding the molecular mechanism of Aβ metabolism modulated by NMDA receptors. Furthermore, it demonstrated a possible multitarget therapeutic approach for Alzheimer´s disease, which might be more effective for this complex neurodegenerative disease [125,126].

Another substance from this group, gambierone, was isolated from the dinoflagellate *Gambierdiscus belizeanus*. Although the sequence of cycles has no similarity to CTX, this compound has the biological activity in the neuroblastoma SH-SY5Y cell line similar to that of CTX-3C but with less intensity. In spite of the isolation of gambierone from a hydrophilic fraction (typical of MTX, see below), this compound induced the shift of the voltage-dependent activation curve to negative and promoted the appearance of Na^+^-currents at hyperpolarized potentials in a similar fashion to CTX-3C, although with much less potency. The small cytosolic Ca^2+^ increase evoked by gambierone in nanomolar concentrations in SH-SY5Y cells was not dose dependent but Na^+^-dependent, probably due to the Na^+^/Ca^2+^ exchange. This was not observed with other CTX analogues [110].

**Figure 3:**
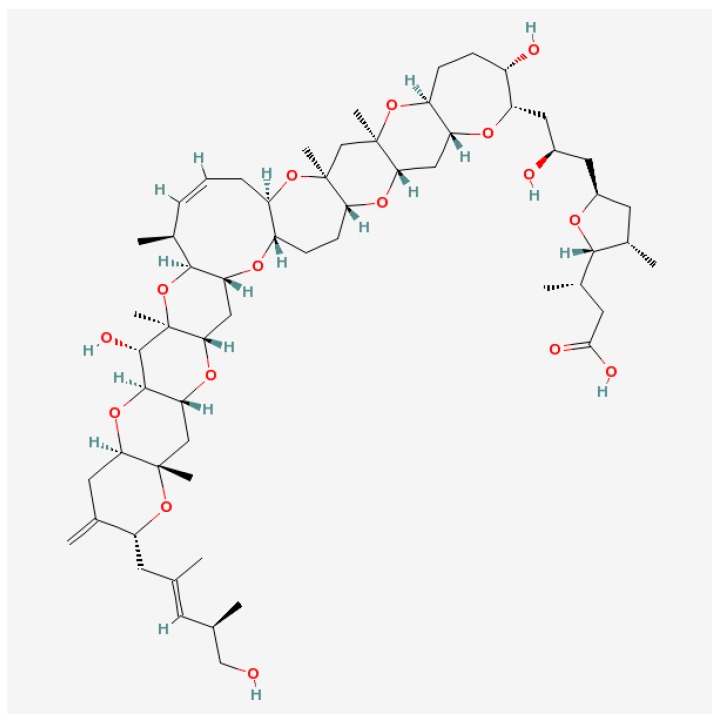
Structure of gambieric acid A [128].

Along with numerous harmful effects, a few but important possibilities of usefulness appear in medicine where marine toxins turned out to be more dangerous for pests, instead of for humans and their animal pets. Nine ether rings, three olefins and one carboxylic acid are characteristic for gambieric acids from *G. toxicus* (Figure 3). The antifungal activity of gambieric acids is extremely potent. They inhibit the growth of *Aspergillus niger* at very low doses. The potency exceeds that of amphotericin-B by 2000-fold. It is probably the most potent antifungals known to date. The toxicity against mice or cultured mammalian cells was moderate, which points to the potential of the acids as antifungal drugs [109]. A convergent synthetic route to the CDEFG-ring (rings marked in alphabetical order) system of gambieric acids A and B with potent antifungal activity against *A. niger* has also been developed [127].

### 5.3. Maitotoxins

**Figure 4:**
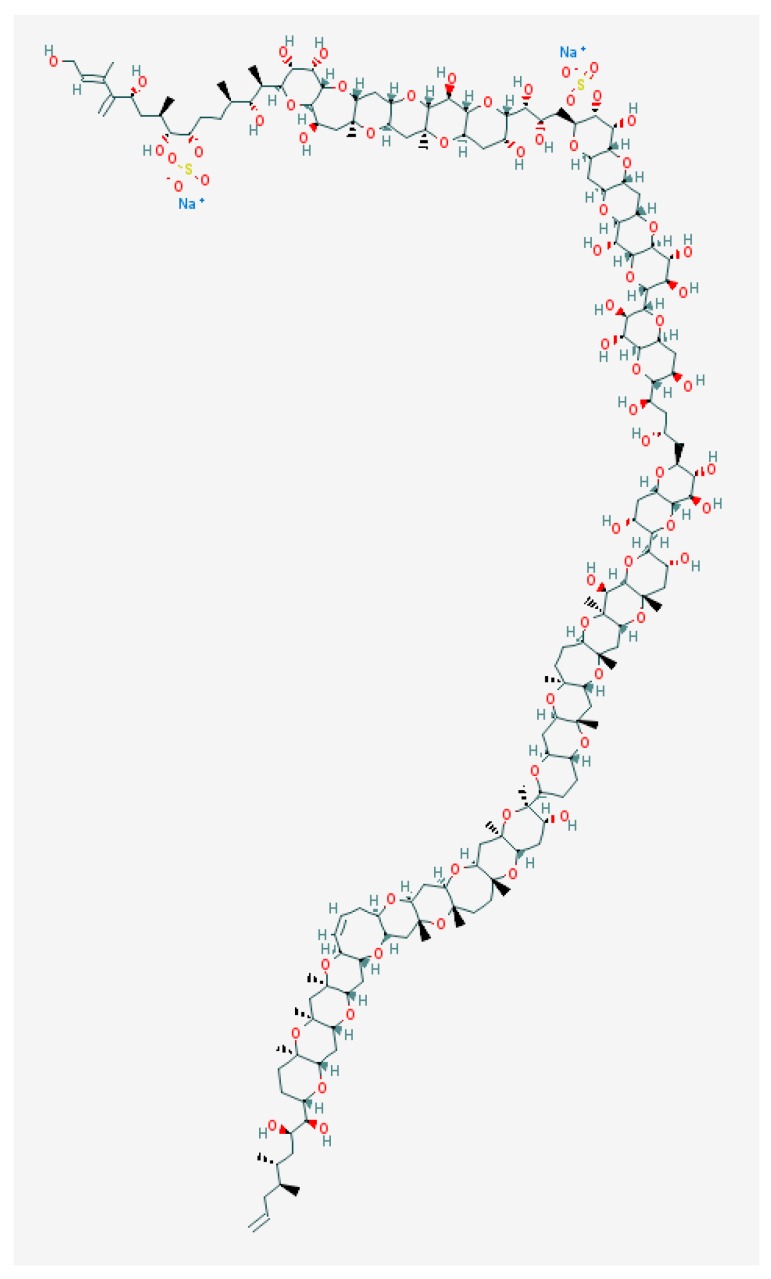
Structure of maitotoxin [129].

Maitotoxin (MTX) differs from other ladder-shape compounds both with regards to molecule size and method of action (Figure 4).

It is one of the largest non-polymeric natural compounds [130,131], constructed of 32 cyclic ether rings with an amphiphilic backbone that contains hydrophobic and hydrophilic regions. One of the MTX regions resembles the CTX-3C backbone by its relatively lipophilic nature, presence of a nine-membered ring and the rigid conformation [83]. However, it is water-soluble and it apparently accumulates in liver, stomach or intestines of fishes [132]. Although MTX-1 accumulation in fish flesh is small, its possible role in pathological CFP symptoms remain highly probable, especially in many Pacific Island nations where eating of non-eviscerated fish is a common practice [57].

Maitotoxins (MTXs) are the most potent marine toxins known to date. The detailed review devoted to various features of maitotoxins was published recently [133]. The main effect of MTX is to induce an inward Ca^2+^-current in various pathways, which has disputable specific variants. The fact that MTX activates cation channels was clear from direct voltage-clamp current measurements (see [133] for review), including voltage-gated Ca^2+^-channels (VGCC) in cultured brainstem neurons [134] and non-selective channels in the cells lacking VGCC [135,136]. MTX, similar to CTX-3C, also affected VGSC by decreasing the peak of Na^+^-current amplitude likely due to inactivation of Na^+^-channels by the rise in Ca^2+^ [83].

Comparing the abilities of various maitotoxin fragments to inhibit the maitotoxin-induced influx of Ca^2+^-ions in rat C6 glioma cells, it was shown that maitotoxin anchors itself in the neuron membrane using its lipophilic domain (i.e., QRSTUVWXYZA′B′C′D′E′F′), which is consistent with Murata’s hypothesis [74]. Maitotoxin presumably binds to the membrane-bound ion channel and opens it, while its hydrophilic domain (i.e., ABCDEFGHIJKLMNOP) remains outside the cell membrane. The latter most likely serves to sequester and facilitate the influx of Ca^2+^ ions into the cell through the channel, once the channel is opened [137].

The methylated MTX fragment of QRSTUVWXYZA′ rings shows a significant growth of the inhibition against leukemia, non-small cell lung cancer, colon cancer, CNS cancer, melanoma, ovarian, renal, prostate and breast cancer [137].

Using the fluorescence Ca^2+^-indicator Indo-1AM to determine the cellular toxic mechanisms of Caribbean isoform of MTX (MTX-C) in insulin-secreting transformed hamster pancreatic islet HIT-T15 cells, a profound increase in free intracellular Ca^2+^ levels was found 3 min after application of 200 nM of MTX-C. Application of MTX-C did not elicit the l-type Ca^2+^-current, although large cationic currents appeared after applying MTX-C to the extracellular solution. However, these cells stably express l-type Ca^2+^-channels [136].

MTX-C causes an opening of non-selective, non-voltage activated ion channels, which permits or elicits further abnormal Ca^2+^-influx. The elevated level of intracellular Ca^2+^ concentration resulting from this Ca^2+^-influx may lead to cellular toxicities. The opening of non-selective cation channels will result in a net positive ion influx due to the higher electrochemical driving force for Na^+^ ions than for K^+^ ions. Such a net Na^+^ influx can depolarize cell membrane potential and activate voltage-gated Ca^2+^-channels in HIT-T15 cells [136].

Ca^2+^ ions are known as “risky messengers” [138] because of their involvement in a huge number of cellular regulatory processes. It is limited by its short effective distance of action [139], but it is compensated by the immense number of diverse binding sites. The pronounced concentration-dependent Ca^2+^-influx evoked by MTX leads to the intracellular acidosis and the cell death in the medium, containing Ca^2+^ ions (i.e., not involving intracellular stores [83]). The rise of acidosis under the action of MTX provides evidence in favor of the activation of Na^+^/H^+^-exchanger. This causes an increase in intracellular Na^+^, a reverse in the operation of the Na^+^/Ca^2+^ exchanger and an influx of Ca^2+^, although the mechanisms underlying the reverse operation of the Na^+^/Ca^2+^ exchanger were not identified [140]. Moreover, it has been recently demonstrated that a decrease in intracellular pH causes toxic Ca^2+^ influx through the co-activation of the Na^+^/Ca^2+^ exchanger and the Na^+^/H^+^ exchanger in neuroblastoma cells [141].

MTXs also interfere in a number of developmental processes that are ruled by coordinated ionic fluxes, especially fertilization [142]. It is known that a rise in intracellular Ca^2+^ levels plays key role in sperm functions in addition to capacitation, motility and acrosomal reaction [142,143]. It is suggested that two different types of Ca^2+^ channels participate in the mammalian sperm acrosome reaction: one is required for a fast transient change in Ca^2+^ levels and another is required to sustain an elevated intracellular Ca^2+^ concentration. The first phase of Ca^2+^ entry is supplied probably by VGCC, while the sustained Ca^2+^ influx may be carried through a store depletion-operated pathway [144,145]. MTX activates the Ca^2+^ influx and induces the acrosomal reaction in mammals. The data initially suggested that the actions of MTX is realized via the same ways as those of other agents that evoke an increase in intracellular Ca^2+^ and triggers the acrosome reaction, including the physiological ligands of the *zona pellucida* (ZP) [146]. However, it was found that the differences exist in the acrosome reaction induced by MTX and the ZP ligands in mammals [147]. The phospholipase C (PLC)-dependent signaling pathway participates in the ZP-induced acrosome reaction. The use of PLC inhibitors blocked the acrosome reaction induced by MTX in mouse but not in human sperm, unveiling species-specific variants of the acrosome reaction induced by the toxin [133]. MTX (3 nM) activates Ca^2+^ entry in rat round spermatids and pachytene spermatocytes incubated in a glucose-containing medium in the presence of an external Ca^2+^. In the presence of lactate, the Ca^2+^ entry activated by MTX in rat spermatids is quite slow compared with the initial rate of MTX-induced Ca^2+^ entry in a glucose-containing medium. MTX-activated Ca^2+^ channels in spermatogenic cells can be regulated by a Ca^2+^-CaM-dependent protein kinase [148].

Along with MTX-1 that is present in *G. toxicus*, the other maitotoxins, MTX-2 and MTX-3, from different Queensland strains of *Gambierdiscus* sp. were described, displaying high levels of toxicity in the in vitro systems [111]. The newly discovered species *G. cheloniae* [29] и *G. lapillus* [30] from Australian waters do not produce MTX-1 or known ciguatoxins’ analogs from microalgae (CTX-3B, 3C, CTX-4A and 4B). However, this species probably produces MTX-3, which is highly toxic for mice at intraplantar injection [29]. *G. lapillus* also is assumed to produce MTX-3 [30]. The analyses of *F. paulensis* toxins brought the first data on the presence of MTX, 54-deoxyCTX-1B and gambieric acid A in this species [53]. The same is true for the newly described *Gambierdiscus honu* that produced the putative MTX-3 analogue, although this species did not produce CTX nor MTX. Extracts of *G. honu* were shown to be highly toxic to mice by intraperitoneal injections [24].

## 6. Compounds, Produced by Karenia and Their Physiological Effects

Furthermore, as the harmful algae of the *Gambierdiscus* lineage, *Karenia* microalgae produce a number of substances that differ in chemical and pharmacological properties. In the present case, true physiological antagonism exists between some of them.

### 6.1. Brevetoxins and Others

Brevetoxins (BTXs) have been implicated in contributing to the morbidity and mortality of marine mammals [6]. While CTXs and MTX are accumulated in fish, BTXs accumulate mainly in shellfish. High levels of BTX and its analogues caused the total closure of all bivalve industries in New Zealand when detected in shellfish samples [61]. Similar to CTXs, BTXs also can accumulate in live fish by dietary transfer. Fish fed with toxic shellfish and *Karenia brevis* cultures remained healthy and accumulated high levels of BTX in their tissues. Repeated collections of BTX-contaminated fishes from Florida waters reveal that the accumulation of BTX in healthy fish occurs in the wild. Concentrations were highest in the fish liver and stomach contents, which increased during and immediately following the bloom. The persistence of BTXs in the fish food chain was followed for 1 year after the *K. brevis* bloom [149]. In humans, BTXs are the causative agents of NSP and asthma-like symptoms through inhalation exposure [150].

**Figure 5:**
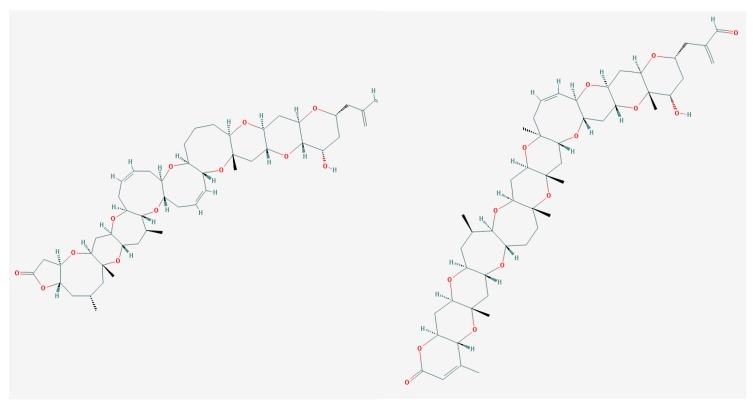
Brevetoxin type A (**left**) and type B (**right**) [151,152].

BTXs consist of 10–11 trans-fused rings, which form 2 variants of skeletal backbones (A- and B-type), and a variety of side chain substituents on the rings distal to the lactone [41,43]. This summarily counts 9 congeners (Figure 5). Type A includes the brevetoxins 1, 7 and 10, while type B includes brevetoxins 2, 3, 5, 8 and 9 [41].

Similar to the above-mentioned CTXs, the molecular target of BTXs is an ion channel containing one or more α-helices, to which polyether ladder-shape compounds bind with extremely high affinities (nanomolar to picomolar K_d_ values). This binding occurs first with VGSC [41,153]. [^3^H]PbTx-3 specifically binds to skeletal muscle (Na_v_1.4) or the cardiac (Na_v_1.5) Na^+^-channel α-subunit isoforms expressed in human embryonic cells (HEK). Na_v_1.5 Na^+^-channels appeared to overall be less sensitive to BTXs compared with the Na_v_1.4 isoform. BTXs type A (PbTx-1) and type B (PbTx-3 and PbTx-2) target both cardiac and muscle channels. BTXs type B exhibit a lower affinity for the heart compared to the skeletal muscle channel. PbTx-1 was 5- and 3-fold more potent than PbTx-3 on HEK-hH1a and HEK-μ1 cells, respectively. Neither PbTx-1 nor PbTx-3 added alone resulted in the reduction in the viability of transfected HEK cells [154].

The evaluation of the relative affinity of PbTx-2 and -3 as well as CTX with native VGSCs was performed by competitive binding in the presence of [^3^H] PbTx-3 in the brain, heart and skeletal muscle of rat and the marine teleost fish *Centropristis striata*. No differences between the rat and fish were observed in the binding of PbTxs and CTX to either brain or skeletal muscle. However, [^3^H] PbTx-3 showed a substantially lower affinity for rat heart tissue, while it bound with the same affinity to heart as to brain or skeletal muscle in the fish [79].

Neurons from the primary cell cultures of freshwater turtle *Trachemys scripta* exposed to 100–2000 nM of PbTx-3 show a dose-dependent decrease in cell viability. PbTx-3 evoked a significant Ca^2+^ influx in these cells, which could be fully abrogated by the VGSC antagonist tetrodotoxin, NMDA receptor blocker MK-801 and tetanus toxin. This indicates that the mode of action in turtle neurons is the same as in mammalian cells [155].

BTX also evokes embryostatic effects and failures of differentiation in the medaka *O. latipes* [156,157,158] and in the larvae of the northern quahog, *Mercenaria mercenaria*; eastern oyster, *Crassostrea virginica*; and bay scallop, *Argopecten irradians* [159]. No direct data exist with regards to the coupling of these effects with the activity of ion channels, although it is the most probable mechanism.

Toxins of *K. mikimotoi* and *K. papilionacea* were not identified until recently [160]. The presence of PbTx or PbTx-like compounds in other Kareniacea, including *K. papilionacea*, was suggested [161,162,163]. It was not confirmed by LC/MS for *K. papilionacea, K. brevisulcata, K. concordia, K. cristata* and *K. selliformis* [37,161,162,163,164]. However, it was later established that similar to *K. brevis* dinoflagellate, *K. papilionacea* produces brevetoxin-2 (PbTx-2). Toxin production in *K. papilionacea* increased in response to hypo-osmotic stress, as previously observed in *K. brevis* [165].

*K. mikimotoi* produces several classes of compounds, including gymnocins, hemolytic glycolipids and polyunsaturated fatty acids, which have cytotoxic, hemolytic and ichthyotoxic properties [166]. Although BTXs were not found in *K. mikimotoi*, other substances produced by this microalga are thought to cause mortality in fish and invertebrates. These other substances include hemolytic and cytotoxic compounds gymnocin A and B in addition to a mixture of liposaccharides and lipids [33,167,168,169]. *K. mikimotoi* and *K. papilionacea* produces ichtiotoxic polyunsaturated fatty acids (PUFA), such as eicosapentaenoic acid, and hemolytic toxins that decrease the survival rate of the grazers [170,171,172]. The PUFA produced by *K. mikimotoi* were toxic for various tissue cultures, decreased bacterial bioluminescence and were allelopathic to various algae as it disrupted their cell membranes [173,174] (see also [66] for review). High levels of superoxides with allelopathic activity to other microalgae and bacteria were also found to be produced by *K. mikimotoi* [175,176,177].

The microalgae *Karenia* together with the BTXs also produces gymnodimines (GYMs) [37,178,179] and less toxic gymnocine (see [180]).

**Figure 6:**
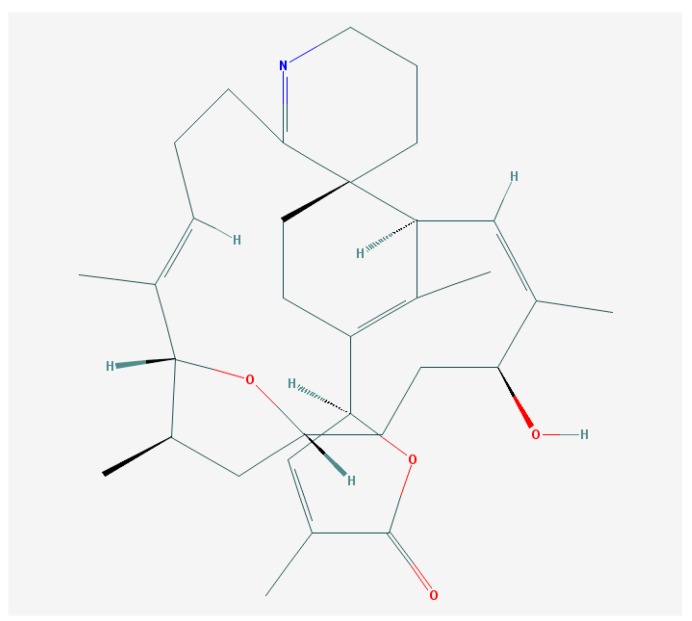
Structure of gymnodimine A [184].

**Figure 7:**
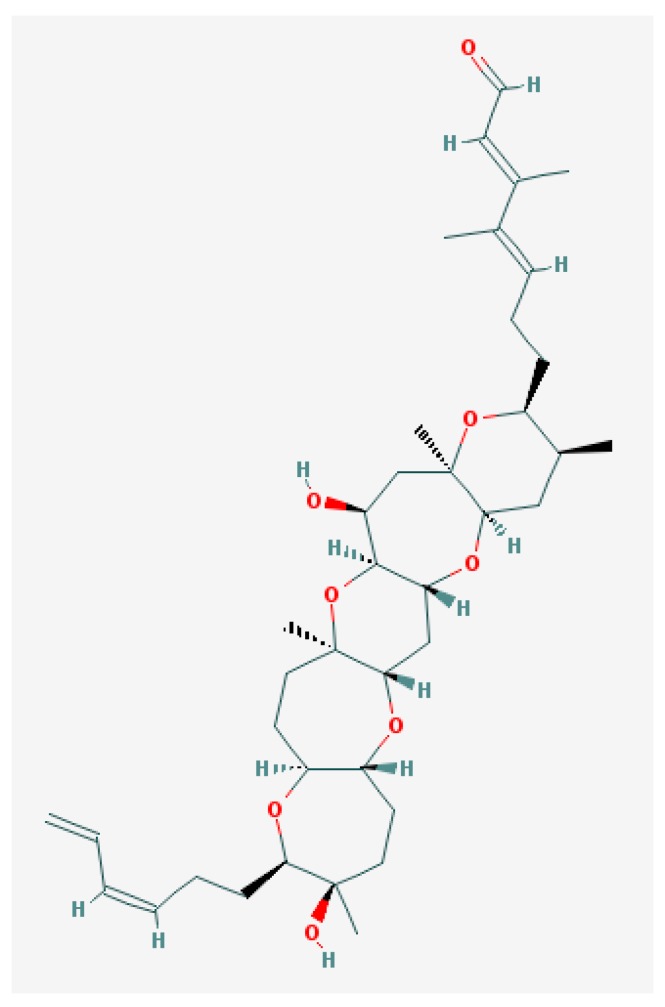
Brevenal structure [193].

GYMs (Figure 6), isolated from extracts of the planktonic dinoflagellate *Karenia selliformis* from the Gulf of Mexico [37], possesses a pentacyclic structure (Figure 7; spiro center including a spirocyclic imine ring system [35]; see also [181]). The next two analogues from this group, GYM-B and GYM-C, were discovered in cell cultures of the toxic phytoplankton producer from the coast of New Zealand [182]. Gymnodimine A and its analogues reversibly blocks nicotinic acetylcholine currents especially in homomeric human α7 nicotinic acetylcholine receptors (nAChRs) expressed in *Xenopus* oocytes. It is probably the main cause of the acute symptomatology, including sudden death observed during the mouse bioassay [183].

Finally, the direct interaction between GYM-A and muscle type nicotinic AChRs (nAChRs) was demonstrated by the concentration-dependent displacement of radio-labeled α-bungarotoxin (a peptidic antagonist) in competition binding experiments performed in HEK293 cells expressing muscle nAChRs [183,185]. At the same time, this compound has no effects on muscarinic AChRs [186].

With regards to the data existing on the decrease in β-amyloid peptide (Aβ) levels in cortical neurons by nAChR antagonists [187], the effect of gymnodimine was studied. This also demonstrated the decrease in the intracellular Aβ accumulation and the levels of the hyperphosphorylated isoforms of the tau protein [186]. Such an effect is suggested to be mediated by the increase in the inactive isoform of the glycogen synthase kinase-3, the decrease in the levels of the active isoform of the ERK1/2 kinase and the increase in ACh synthesis [125]. At the same time, gymnodimine had no effect on the expression of several signal transduction proteins (c-Jun, ATF-2, ATF-3) [186].

Other species in the family Kareniaceae that are known to produce toxins and affect fish/shellfish include *K. brevisulcata* and *K. selliformis*. *K. brevisulcata* is responsible for the mass poisoning of marine life around the southern coast of the North Island of New Zealand from January to March 1998 [34,188], while *K. selliformis* produces the shellfish toxin GYM [182,189,190]. *K. brevisulcata* produces two novel classes of toxins. Firstly, these are ten high molecular weight polycyclic ether lipid-soluble toxins (KBTs). KBT-F and KBT-G are strongly hemolytic and cytotoxic in P388 and Neuro2a cells in addition to being highly toxic intraperitoneally to mice. They have some structural similarities to gymnocins, although KBTs are larger molecules and more toxic than gymnocin-B [162]. Secondly, six water-soluble brevisulcatic acids (BSXs), which are VGSC agonists with some structural similarities to brevetoxin-A were detected. At the same time BTXs were not found in this species. BSX-4 and BSX-5 are proposed to be the primary toxins produced by the cells, which are hydrolyzed to the open-ring compounds when released from the cells to the culture medium. However, the structures have not yet been fully elucidated due to conformers confounded by the NMR [38].

Sets of toxins from the other species *K. asterichroma* and *K. umbella* still remain uncovered. The potentially toxic *K. papilionacea* and *K. umbella* have been associated with fish kills in New Zealand and Australia [37,191,192]. However, their fish-killing mechanism remains to be determined. Further work is required to determine the toxic mechanism for several gymnodinioid species, particularly because these dinoflagellates often occur in mixed Kareniaceae blooms in which their species identification is difficult. The possible synergistic effect of mixed blooms on fish also requires attention [154].

### 6.2. Brevenal—Dissident Ladder-Shape Compound

Within a wide spectrum of ladder-shape compounds, brevenal is produced by Florida’s red tide dinoflagellate *Karenia brevis* and is separated by its properties (Figure 7).

It is the non-toxic natural product competing with tritiated BTX for site 5, which is associated with the VGSCs of rodent brain. Brevenal, obtained from either laboratory cultures or field collections, protects fish from the neurotoxic effects of BTX exposure [194]. It contains only five polyether rings and binds to the VGSC at the specific “brevenal site”, which is distinct from the BTX site 5 [195]. Brevenal successfully displaces [^3^H]-PbTx-3 from the receptor site 5 of VSSC [194]. The radioactive analog of brevenal ([^3^H]-brevenol) was produced. It binds with K_D_ of 67 nM to VGSC of rat brain synaptosomes. Brevenal and brevenol competed for [^3^H]-brevenol binding with K_i_ values of 75 nM and 56 nM, respectively [195].

As brevenal and brevenol were able to completely inhibit the specific binding of [^3^H]-PbTx-3 to site 5 of VSSCs, it was assumed that brevenal and brevenol were simply two additional site 5 ligands. However, neither PbTx-2 nor PbTx-3 were able to inhibit [^3^H]-brevenol binding. These results suggest that [^3^H] brevenol and by inference, brevenal, bind to a site (i.e., the “brevenal site”) that is distinct from site 5 on VGSCs. As brevenal and brevenol can completely inhibit the [^3^H]-PbTx-3 specific binding, the brevenal site is obviously associated with site 5 in some manner [195].

Brevenal also acts as a functional antagonist for ciguatoxin and protects against P-CTX-1B-induced lethality in fish. Moreover, brevenal is a powerful antagonist of PbTx-2-induced Ca^2+^ influx in neurons in a similar way to gambierol [196]. As PbTx and P-CTX-1B share the same binding receptor-site 5 on VGSC [197], brevenal is therefore a potential inhibitor for CTXs, as it inhibits BTX binding to site 5 of VSSC [88]. This forms the reason for brevenal being a lead compound for the development of treatment for cystic fibrosis, neurotoxic shellfish poisoning and ciguatera, which involve VGSC activation [89,194,198].

P-CTX-1B also promotes catecholamine secretion from bovine chromaffin cells, an effect that is insensitive to concomitant activation of capacitive Ca^2+^ entry. Brevenal significantly blocked P-CTX-1B-induced catecholamine secretion. This effect is partially reversible [90]. Importantly, neither of these two inhibitors affected the catecholamine basal release level or nicotine-induced catecholamine secretion [89].

The function of brevenal in the dinoflagellate thylakoid membrane of *K. brevis* considered in the next section may explain such unusual physiological characteristics of this compound. Interestingly, the combination of brevenal/brevetoxin in *K. brevis* has an analogous ciguatoxin/gambierol couple in *Gambierdiscus*.

## 7. Possible Intrinsic Functions of Ladder-Shaped Toxins in Microalgae

A number of studies and basic reviews have been published on the extrinsic role of BTX and CTX as ligands of ion channels. However, very little research has been conducted on the intrinsic functions of these substances in the organisms producing them. For a long time, hypotheses in this field remained quite obvious. The basic one suggested that the microalgae produces the toxins as a deterrent against ingestion, which is consistent with a classic defensive role for these neurotoxins [199,200,201]. Seemingly, no one noticed that a great variety of BTXs and CTXs as well as the largest non-polymeric organic maitotoxin molecule are assigned by nature exclusively to prevent the grazing of *Karenia* and *Gambierdiscus* microalgae. It is akin to building the Great Pyramid on the grave of a beloved hamster.

In fact, such strange uneconomical phenomena of the living organisms filling the environment with various sophisticated chemicals are not considered to be solely exotic. For example, the serotonin-like substance leaks from the suspension of unfertilized sea urchin eggs to the outer medium, although it is the transmitter that has multiple functions in various early embryos [202,203].

Recently a new approach was published, which suggests that the original function of BTXs is associated with the regulation of intracellular ionic homeostasis when salinity levels change in coastal sea waters [204,205,206]. Since the production of BTXs is significantly increased under the influence of osmotic shock, the authors suggested that algae cells can react through the activation of Na^+^-channels to sharp fall of the salinity and correspondingly, the correction of the intracellular ionic balance. However, more precise experiments carried out simultaneously in three labs denied this possibility [201] and returned the researchers to their initial position—to the idea of microalgae self-defense against grazing by zooplankton [41].

**Figure 8:**
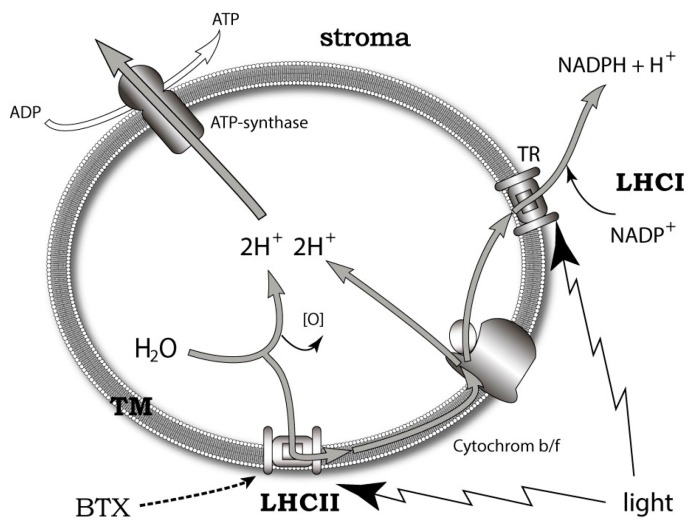
Simplified scheme of thylakoid membrane photosystems—a possible target for brevetoxins. In this diagram, TM—thylakoid membrane, LHCI and LHCII—light-harvesting complexes and TR—thioredoxin.

A more promising approach to the problem was developed on the basis of the data obtained using the new fluorescent and photoactivatable BTX derivatives. It was shown that these compounds localize in the lipophilic thylakoid membrane of *K. brevis* chloroplast, where it binds to the light-harvesting complex II (LHCII) and thioredoxin. LHCII is important for non-photochemical quenching (NPQ) of the light energy excess (Figure 8). At the same time, thioredoxins maintain a redox homeostasis within the chloroplast and influence the scavenging of reactive oxygen (ROS) [41]. Furthermore, another ladder-shaped toxin of this lineage (MTX) is able to increase ROS levels [207,208]. The difference between NPQ and ROS production in highly and less toxic *K. brevis* strains has also been found. The dependence of LHCII on BTXs decreased at a neutral pH and low levels of light. Interestingly, BTXs also have a significant influence on the ROS levels in *Crassostrea virginica* gametes by somewhat increasing them in the sperm, while approximately dropping these levels by 3-fold in oocytes [209].

Based on the above-mentioned points, the conclusion was made that the localization of brevetoxin to chloroplast and the identification of a brevetoxin receptor in *K. brevis* could implicate an endogenous role for the BTXs and other polyether ladders. The exact mechanisms of such a modulation remain unclear. There seem to be three possibilities: (1) BTX interacts with LHCII directly, inducing conformational changes when associated with NPQ; (2) BTX activates ion channels in thylakoid membrane; or (3) self-assembly of BTX into transmembrane pores, triggering the cation current via the thylakoid membrane [41].

The molecules of polyether ladder-shape toxins are known to migrate onto a cell membrane where they interact with membrane proteins. It was also shown that BTXs aggregate into artificial lipid bilayers [208]. The results obtained in *K. brevis* [41] are consistent with the data on the localization of another dinoflagellate toxin (okadaic acid [209]) in the chloroplast and the toxin from cyanobacteria microcystine in the thylakoid membrane of cyanobacteria [210].

**Figure 9:**
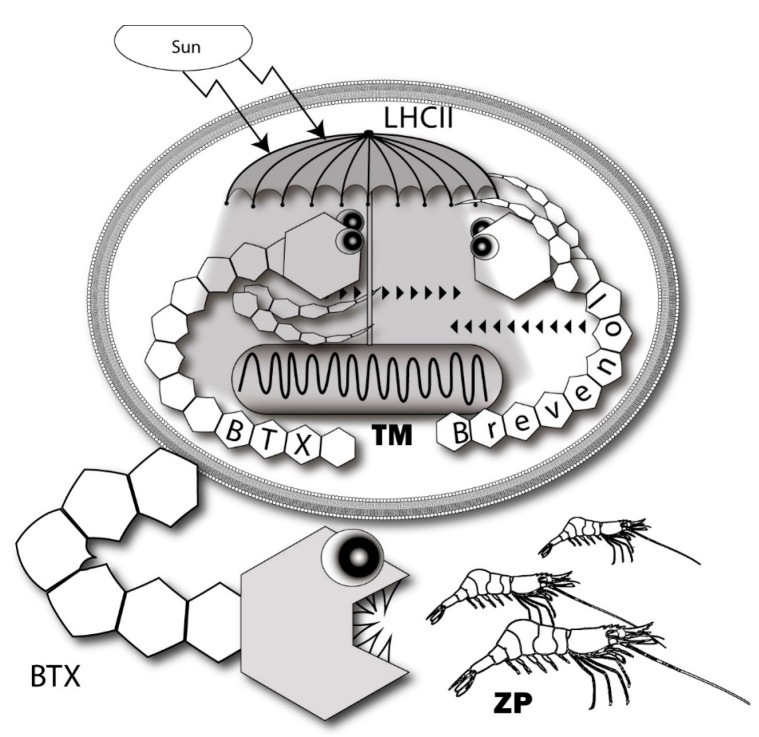
Intrinsic and extrinsic functions of BTXs and brevenol in the thylakoid membrane of *Karenia* microalgae. BTX and brevenol act as antagonists at LHCII intracellularly and BTX functions as a feeding deterrent in the outer medium. In this diagram, BTX—brevetoxin, LHCII—light-harvesting complex II, TM—thylakoid membrane and ZP—zooplankton.

The role of endogenous BTX-antagonists, brevenal and brevenol, are consistent with the idea related to the mechanism of modulating intracellular photosynthetic processes. It requires both positive and negative regulators in contrast to the traditional feeding deterrent concept where it looks absurd [89,194] (Figure 9). Gambierol and gambieric acid A also inhibit the binding of the BTXs to the VGSC [116]. Essentially, components of the analogous scheme of regulation are also present in *Gambierdiscus*. A propos, another ladder-shaped toxin of this lineage—MTX is able to increase ROS levels [211,212].

Thus, it is suggested that the initial intrinsic primary function of polyether ladders was specifically targeted to α-helix transmembrane proteins localized in thylakoid membrane. It was only coincidentally associated with other transmembrane α-helices in higher organisms later, including VGSCs, which have formed the base of widely studied effects nowadays. However, the discovery of the fact that BTXs have intracellular biochemical role associated with photosynthesis does not exclude the possible extracellular function of a feeding deterrent. Indeed, it was shown that secondary metabolites, such as salicylic acid, also function as feeding deterrents as well as hormone and growth regulators in plants [213].

Similarly, it was suggested that the evolutionary initial function of classic neurotransmitters, such as catecholamines and serotonin, is the regulation of intracellular processes, such as protein synthesis and cell cycle control. Such transmitters are the derivatives of substantial amino acids, limiting protein synthesis in animal cells, and can serve as the intracellular probe for the levels of these amino acids inside the cell. Following this, these signal substances obtained the synaptic transmitter function that co-exists with various embryonic and postembryonic ones (such as acting as a local hormone) [103].

## 8. Conclusions

Thus, multiple ladder-shaped compounds produced by the dinoflagellates *Gambierdiscus* sp. and *Karenia* sp. are able to influence practically all mechanisms of cellular ionic balance in various ways, including VGSC, voltage-gated K^+^-channels and voltage-gated Ca^2+^-channels. Furthermore, there is preservation of the pronounced specificity of every individual compound concerning this or that regulation mechanism.

Human anthropocentrism forms an attitude to the observed phenomena based on its practical implications, its usefulness or harmfulness. This forms the reason for marine toxins becoming interesting first for medical reasons, including both therapeutic and epidemiological studies. Future promising directions would involve studies of the mechanisms of pathologies and use of ladder-shape polyethers as drugs for neurodegenerative diseases, such as Alzheimer syndrome, or potent antifungal agents. However, the most intriguing research would probably be devoted to the intrinsic functions of these compounds and their respective biological meaning. The medical aspects only reflect functions of mechanisms underlying housekeeping processes of marine microalgae. It may have nothing in common with human health and is implemented through participation of substances occasionally toxic to external organisms, although these possess some important functions in the algal cells themselves. These very intrinsic functions have to become the main subject of research, because functions of such sophisticated substances surely have significant importance for cells and belong to basic conservative mechanisms explaining the evolution of living beings.
